# Immunobiotics for the Bovine Host: Their Interaction with Intestinal Epithelial Cells and Their Effect on Antiviral Immunity

**DOI:** 10.3389/fimmu.2018.00326

**Published:** 2018-03-02

**Authors:** Julio Villena, Hisashi Aso, Victor P. M. G. Rutten, Hideki Takahashi, Willem van Eden, Haruki Kitazawa

**Affiliations:** ^1^Laboratory of Immunobiotechnology, Reference Centre for Lactobacilli (CERELA-CONICET), Tucuman, Argentina; ^2^Immunobiotics Research Group, Tucuman, Argentina; ^3^Food and Feed Immunology Group, Laboratory of Animal Products Chemistry, Graduate School of Agricultural Science, Tohoku University, Sendai, Japan; ^4^Cell Biology Laboratory, Graduate School of Agricultural Science, Tohoku University, Sendai, Japan; ^5^Livestock Immunology Unit, International Education and Research Center for Food Agricultural Immunology (CFAI), Graduate School of Agricultural Science, Tohoku University, Sendai, Japan; ^6^Department of Infectious Diseases and Immunology, Faculty of Veterinary Medicine, Utrecht University, Utrecht, Netherlands; ^7^Laboratory of Plant Pathology, Graduate School of Agricultural Science, Tohoku University, Sendai, Japan; ^8^Plant Immunology Unit, International Education and Research Center for Food Agricultural Immunology (CFAI), Graduate School of Agricultural Science, Tohoku University, Sendai, Japan

**Keywords:** immunobiotics, antiviral immunity, beneficial microbes, bovine rotavirus, toll-like receptor 3 pathway, inflammation, agricultural immunology

## Abstract

The scientific community has reported several cases of microbes that exhibit elevated rates of antibiotic resistance in different regions of the planet. Due to this emergence of antimicrobial resistant microorganisms, the use of antibiotics as promoters of livestock animals’ growth is being banned in most countries around the world. One of the challenges of agricultural immunology therefore is to find alternatives by modulating the immune system of animals in drug-independent safe food production systems. In this regard, in an effort to supplant antibiotics from bovine feeds, several alternatives were proposed including the use of immunomodulatory probiotics (immunobiotics). The purpose of this review is to provide an update of the status of the modulation of intestinal antiviral innate immunity of the bovine host by immunobiotics, and the beneficial impact of immunobiotics on viral infections, focused on intestinal epithelial cells (IECs). The results of our group, which demonstrate the capacity of immunobiotic strains to beneficially modulate Toll-like receptor 3-triggered immune responses in bovine IECs and improve the resistance to viral infections, are highlighted. This review provides comprehensive information on the innate immune response of bovine IECs against virus, which can be further investigated for the development of strategies aimed to improve defenses in the bovine host.

## Introduction

Over the past decades, the global bovine production has been subjected to intensification, in order to improve efficiency of production because of the demand from a growing human population. The intensification of bovine production involved the application of confinement methods characterized by the concentration of animals in large outdoor feedlots or in specialized indoor environments. In confinement, the potential for transfer of pathogens among animals is higher, as there are more animals in a smaller space (1–3).

Severe gastrointestinal infectious diseases causing malabsorption and diarrhea are important causes of discomfort and death in young calves, resulting in important economic losses to bovine producers. Gastrointestinal infectious diseases are able to cause significant economic losses to the cattle industry in big cattle-producing countries and can impair the development of cattle industry in small cattle-producing countries (1–3). In particular, the neonatal gastroenteritis in the bovine host is a multifactorial disease. This disorder can be caused by different bacterial or viral pathogens, including bovine coronavirus (BCV), bovine rotavirus (BRV), and bovine viral diarrhea viruses (BVDV) (4, 5). Although these viral pathogens belong to distinct families and possess different physical characteristics, they are all able to infect intestinal epithelial cells (IECs), generate villous atrophy, and cause inflammatory intestinal tissue damage and diarrhea.

Probiotics are defined as live microorganisms with the capacity to confer a health benefit on the host when administered in adequate amounts. Among them, those that are able to impact on human and animal health by modulating the mucosal and systemic immune systems have been called immunobiotics. It has been reported that immunobiotic lactic acid bacteria are able to generate protection against viral pathogens by differentially modulating antiviral immune responses in humans and livestock animals like pigs (6, 7). It is also believed that immunobiotics could be used in cattle feeds to improve bovine health and produce safe animals (8–10).

The purpose of this review is to provide an update of the status of the modulation of intestinal antiviral innate immunity in the bovine host by immunobiotics, and their beneficial impact on viral infections. The results of our group, which demonstrate the capacity of immunobiotic strains to advantageously modulate Toll-like receptor (TLR)-3-triggered immune responses in bovine IECs and improve the resistance to viral infections, are particularly highlighted.

## The Use of Probiotics in the Bovine Host

Before weaning, dairy calves are highly susceptible to infectious diseases. For several years, antimicrobial compounds have been used to reduce the severity and mortality of infectious diseases and to improve economic benefits in terms of enhanced bovine performance and diminished medication expenses. However, the use of antibiotics in livestock animal management is in question because of the enhanced resistance of microbes to antimicrobial compounds. Therefore, there is an urgent need to reduce and finally eliminate the use of antibiotics in livestock and for this purpose many feed additives have been proposed including beneficial microbes (8–10). In fact, research from the past decade has provided evidence that probiotic bacteria and prebiotics can be effectively used to improve health and growth in calves and reduce the use of antibiotics (Table 1), although detailed mechanistic studies were not performed. The production of antimicrobial compounds, inhibition of adherence or aggregation with pathogens and the modulation of the microbiota were described as mechanisms of probiotics action in the bovine host [reviewed in Ref. (10)]. Immunomodulation was also proposed as a mechanism of bovine probiotics as mentioned later.

**Table 1:** Probiotics for the bovine host.

Strain	Viability	Route	Host	Effects	Reference
*Lactobacillus acidophilus* LAC-300	Viable	Oral	Holstein calves	Increase in body weight gain. Improvement in fecal scores	(11)
*Bifidobacterium pseudolongum* M-602	Viable	Oral	Holstein calves	Increase in body weight gain. Improvement in fecal scores	(11)
*Bifidobacterium thermophilum* S-501, *Lactobacillus acidophilus* LAC-300, and *Enterococcus faecium* FA-5	Viable	Oral	Holstein calves	No effect on body weight gain was observed. Reduction of diarrhea	(11)
*Saccharomyces cerevisiae, Lactobacillus acidophilus, Bifidobacterium bifidum, Streptococcus termophilus*, and *Aspergillus niger*	Viable	Oral	Holstein calves	Improvement in daily live weight gain and feed efficiency ratio. Reduction of diarrhea	(12)
*Lactobacillus rhamnosus* GG	Viable	Oral	Holstein calves	The probiotic strain survived the gastrointestinal transit. No beneficial effect was recorded	(13, 14)
*Saccharomyces boulardii*	Viable	Oral	Holstein bull calves	Treated animals consumed more grain, had increased weight gain, and increased plasma glucose concentrations. Days with diarrhea were reduced	(15)
*Lactobacillus acidophilus* 15	Viable	Oral	buffalo calve (1 day to 31 weeks)	Weight gain was improved and feed: gain ratio was reduced	(16)
Multispecies probiotic preparation: *Lactobacillus acidophilus* W55, *Lactobacillus salivarius* W57, *Lactobacillus paracasei* W56, *Lactobacillus plantarum W59, Lactococcus lactis* W58, and *Enterococcus faecium* W54	Viable	Oral	Male Holstein-Friesian calves	Probiotics enhanced growth rate and average daily gain and feed efficiency were significantly improved. Modest effect on diarrhea	(17)
Calf-specific multistrain probiotic preparation (six lactobacilli strains)	Viable	Oral	Male Holstein-Friesian calves	Probiotics enhanced growth rate and average daily gain and feed efficiency were significantly improved. Treatment reduced the incidence of diarrhea and the fecal counts of coliforms	(17)
*Lactobacillus casei* subsp. *casei* JCM1134^T^ and Dextran	Viable	Oral	Holstein dairy calves	Increase the milk production	(18)
Lactobacillus *acidophillus, Bifidobacterium bifidum*, and *Enterococcus faecium*	Viable	Oral	Holstein calves	Significant reduction of diarrhea but no effect on mastitis	(19)
*Bacillus subtilis* natto	Viable	Oral	Male Holstein calves	Increased average daily gain and feed efficiency. No difference in serum IgE, IgA, and IgM, whereas serum IgG and IFN-γ were higher in probiotic-treated than in the controls.	(20)
*Lactobacillus plantarum* 220, *Enterococcus faecium* 26, and *Clostridium butyricum* Miyari	Viable	Oral	Holstein bull calves	Increased the numbers of CD282^+^ monocytes, CD3^+^ T cells and CD4^+^, CD8^+^, and WC1^+^ γδ T cell in blood. Increment of production of IL-6, INF-γ, and TNF-α were also observed	(21)
*Faecalibacterium prausnitzii* 34, *Faecalibacterium prausnitzii* 35, *Faecalibacterium prausnitzii* 1S, and *Faecalibacterium prausnitzii* 2S	Viable	Oral	Preweaned dairy Holstein heifer calves	Decreased the incidence of severe diarrhea and related mortality rate, while increasing weight gain	(22)
*Lactobacillus plantarum* GF103	Viable	Oral	Male Holstein calves	No significant differences were observed in dry matter intake or average daily gain, but the feed conversation ratio was improved. Treatment improved mitogen-induced lymphocyte proliferation	(23)
Kefir	Viable	Oral	Female Holstein calves calves	Kefir intake improved fecal scores and reduced days with diarrhea during the first 2 weeks of life. No effect on weight gain	(24)
Milk fortified with symbiotic complex containing prebiotics (mannan-oligossacharides) and probiotics (*Lactobacillus acidophilus, Enterococcus faecium, Bacillus subtilis, Saccharomyces cerevisiae*)	Viable	Oral	Female Holstein heifer calves	Symbiotic did not affect weight gain or feed efficiency of calves but it improved fecal scores	(25)

As described for other livestock animals, the most critical stage in the bovine life is the period from birth to weaning. During this stage factors like nutrition can directly affect the immune system development and function and impact later in bovine performance (26). However, nutritional interventions during this phase are often inappropriate due to the elevated costs of milk feeding. The administration of poor-quality milk or colostrum, and the additions of antibiotics are common practices. Poor nutrition during preweaning stage often conduces to low weaning weight and impaired immunity, thereby increasing losses related to disease. Therefore, the majority of research studying the influence of beneficial microbes in the bovine host has been performed during this period of life.

Early studies showed that orally administered *Bifidobacterium pseudolongum* M-602 or *Lactobacillus acidophilus* LAC-300 enhanced the gain of body weight and reduced diarrhea frequency in calves (11). Similarly, several subsequent studies reported that treatments with probiotic microorganisms beneficially influence body weight gain, body height, milk production, and the general health condition of calves (Table 1). Additionally, oral treatment with probiotics significantly reduced the incidence and the severity of gastrointestinal infections. Moreover, some studies have also shown that probiotic treatments are able to improve not only mucosal defenses in the bovine host but systemic immunity as well (20, 21).

Classical probiotic strains have been used to evaluate their beneficial effects on the bovine hosts including *Lactobacillus, Bifidobacterium, Enterococcus, Bacillus*, and the yeast *Saccharomyces* (Table 1). More recently, new species of beneficial bacteria have been also tested as next-generation probiotics. In this regard, the obligate anaerobic, Gram-positive microorganism *Faecalibacterium prausnitzii* that belongs to the phylum *Firmicutes* has been tested as a potential probiotic for the bovine host (27). Oikonomou et al. (27) reported that the high relative abundance of this bacterium in the first week of life of Holstein calves was associated with improved weight gain and diminished occurrence of diarrhea. Recently, Foditsch et al. (22) confirmed the safety and efficacy of *F. prausnitzii* in young dairy heifers. Researchers reported that the oral administration of viable *F. prausnitzii* reduced severe diarrhea incidence and its related mortality rate. Moreover, *F. prausnitzii* treatment significantly enhanced the weight gain.

The anti-inflammatory properties of *F. prausnitzii*, including its ability to synthetize butyrate, were proposed as factors involved in the beneficial effects observed in calves. These works demonstrated that this intestinal bacterium could be a novel approach to enhance the intestinal health in calves and improve their body weight gain (22).

These studies indubitably show the potential of beneficial microbes to differentially modulate weight gain, intestinal hemostasis, and immunocompetence in young calves. However, the cellular and molecular interactions of probiotics with the cells of the bovine host have not been studied in depth. A better molecular understanding of how the selected beneficial microbes improve resistance against infections by antagonizing pathogens and/or modulating the immune system is needed.

## Bovine IECs as a Model to Study Antiviral Immunity

It is considered that an important step forward toward the understanding of the cellular and molecular interactions of pathogenic or probiotic microorganisms with the bovine host is the establishment of appropriate *in vitro* systems models. Therefore, the development of suitable bovine cell cultures such as IECs would be of great value to advance in this field of research. Those cell cultures should be minutely characterized with regard to their permissiveness for bacterial and viral adhesion and invasion, and the ability to sense microbial-associated molecular patterns through pattern recognition receptors (PRRs) (28).

Primary cultures of small or large IECs have been used to evaluate the effects of microbial virulence factors, toxic compounds, and antimicrobial factors in cattle. Moreover, those primary cultures have been also used for the study of innate immune responses through PRRs signaling (28–33). Soft mechanical agitation combined with enzymatic digestion using dispase and collagenase has been proved to successfully release viable intact bovine colonic cells. However, these cells suspensions contained contaminating non-epithelial cells (mostly fibroblasts) and therefore, a series of purification steps was required to obtain relatively pure bovine colonic cells. The development of primary bovine cell lines from rectum, colon and ileum was reported by Dibb-Fuller et al. (29). Those bovine primary cell cultures were successfully used to evaluate the interaction of several intestinal pathogenic bacteria with bovine IECs and to determine mechanisms of adherence and invasion. More recently, Zhan et al. (34) successfully cultured primary bovine IECs and established a novel clone cell method. Authors demonstrated the expression of E-cadherin and cytokeratin 18, as well as characteristics of epithelial-like morphology in this new cell line. However, the immunological characteristics of cells or viral infections have not been evaluated in those systems.

As mentioned earlier, viral infections in livestock animals could cause a fatal disease that implicate serious economic loses. Therefore, the effective and non-costly control of this type of infections is a key factor for improving animal production. It is believed that the clear and detailed understanding of viral pathogenicity as well as host immune response in the bovine host is necessary to develop strategies capable of reducing infectious disease caused by these viruses. In this sense, a deeper understanding of the molecular interactions of virus with bovine IECs is necessary for the development of better prevention strategies to improve protection in animals.

Bovine virus pathology and immune response have been studied mainly in heterologous systems including mouse models (35) and human cell lines (36). Taking into consideration the differences in viral strains, the specific receptors for virus uptake, the factors required for viral replication and pathogenesis as well as the specific species variations in innate immune responses; the information generated in those heterologous models may not be fully applicable to cattle. Therefore, scientists have tried to establish bovine systems for the study of viral infections. One of the earliest works able to confirm that cultured bovine IECs were susceptible to BRV infection was reported by Kaushik et al. (37). Epithelial cultures obtained from jejunal and ileal tissues were incubated with BRV and both cell types were similarly infected with the viral pathogen. Long incubation times of BRV with the epithelial cultures coming from jejunal and ileal tissues resulted in extensive cellular damage and reduced cell viability, which is in line with the knowledge that BRV is a lytic virus. Furthermore, BRV particles were recovered from the culture supernatants confirming that viral replication occurred in bovine IECs (37). However, the immune response was not studied. In addition, those epithelial cell cultures contained fibroblasts, and therefore if the immune response is evaluated in this system it cannot be discriminated whether the response (cytokine production, for example) is mediated by one or both cells, especially considering that the authors also demonstrated that BRV infected and replicated in fibroblasts (37).

Bovine primary IEC cultures have been of value to study the molecular mechanisms involved in diseases caused by pathogens. However, the cellular and molecular interactions of beneficial or commensal microorganisms with bovine IECs cells have been less examined. In addition, the intestinal cell lines established from adult cattle may have limitations in the study of infections with BRV, BVDV, or BCV, since these viruses infect IECs in the gut of young calves (4, 5).

In order to understand: (i) the pathogenesis of bovine viral infections and the subsequent gastrointestinal diseases, (ii) the role of bovine IECs in the generation of mucosal immune responses, and (iii) the effect of beneficial microbes that may be used to advantageously modulate the antiviral immune response in bovine IECs; we have developed an immortalized bovine IEC line from young calves: bovine intestinal epithelial (BIE) cells (38).

Bovine intestinal epithelial cells have an epithelial-like morphology and they grow forming a monolayer with cells that establish close contact between them (38). Scanning electron microscopy analysis revealed that 3-day-old BIE cells have microvilli-like structures on their surface that are irregular and slender. These cellular structures increase in complexity as the cells grow as observed in 10-day-old BIE cells (38). The evaluations of the expression of cytokeratin and specific villin protein, which are known as markers of epithelial cells, have demonstrated that BIE cells are strongly positive for both proteins. In contrast, vimentin and desmin that are markers for mesenchymal cells and muscle cells, respectively, were not found in BIE cells.

Bovine intestinal epithelial cells also expressed the cell-to-cell adhesion molecules ZO-1 and beta-catenin (39). Both proteins were strongly positive in the cell-to-cell contact region when cells reached confluence. Moreover, the functional integrity of BIE cells gradually increased with time as indicated by studies of TEER and paracellular permeability (38). These results provide clear evidence of the intestinal epithelial nature of BIE cells.

## Immunobiology of Bovine Epithelial Cells

Significant progress has been made in the understanding of both the beneficial and detrimental roles of TLR3 in innate antiviral immune responses in mucosal tissues (6, 40). Therefore, to decipher the exact role of TLR3 in antiviral defenses in IECs is of value to understand the mechanisms that activate and regulate the intestinal immune system of the host. Few studies have been conducted on cattle. Those studies are of importance since the determination of the mechanisms involved in the activation and regulation of TLR3 in bovine IECs could give the scientific basis for the development of efficient preventive or therapeutic strategies for reducing severity and mortality of viral diseases, including oral vaccines and functional feeds. Then, the expression of mRNAs of TLRs was evaluated in BIE cells and it was reported that all the genes for these receptors were expressed in this cell line (41). TLR1, TLR3, TLR4, and TLR6 were strongly expressed while TLR5, TLR9, TLR2, and TLR7 were expressed modestly. We were especially interested in expression of TLR3 as the most important receptor detecting double-stranded genomic RNA (dsRNA) from viruses.

Therefore, to confirm these findings, we further examined the expression of TLR3 protein in BIE cells by immunohistochemical analysis and demonstrated that this PRRs is strongly expressed in the cytoplasm of BIE cells (42). Of note, no TLR3expression was detected at the BIE cell surface. Therefore, BIE cells, in addition to displaying characteristics of epithelial cells like those mentioned earlier such as microvilli-like structures, and strong expression of cell-to-cell junctional proteins (38), they also express TLR3 and thus are similar to the IECs of other species.

The innate immune response induced by TLR3 activation in BIE cells was also studied. BIE cells were treated with the TLR3 agonist poly(I:C) and an upregulation of type I interferon (IFN), and proinflammatory cytokines expression was detected. The changes in the expression of inflammatory factors induced by poly(I:C) in BIE cells correlate with the changes reported in various intestinal viral infections of cattle and other hosts. For instance, enhanced gene expression of CCL5 (RANTES), CXCL10 (IP-10), CXCL8 (IL-8), and CCL2 (MCP-1) were observed in rotavirus-infected HT-29 cells (43, 44). In addition, *in vitro* studies showed that the challenge of bovine intestinal tissues with BRV or BCV activated TLR3, upregulated nuclear factor κB (NF-κB) and increase IL-6 production (4). These findings indicate that BIE cells are valuable tools for the *in vitro* study of immune responses mediated by TLR3 in bovine IECs.

Bovine rotavirus is able to induce a potent inflammatory response mediated by IFN and IFN-induced genes as well as inflammatory cytokines. In this regard, studies performed in HT29 cells infected with BRV (A5-13 strain) demonstrated that viral infection significantly upregulated most of the IFN-inducible genes including IL-18, IFN-α-inducible protein 6, IFN-induced transmembrane protein 3, TAP1, DDX58 [retinoic acid inducible gene-I (RIG-I)], and 2′-5′-oligoadenylate synthetase (OAS) 1 as well as several cytokines such as IL-8, CCL5, CXCL10, and CXCL11 (36). Few studies evaluated the BRV infection and its immune responses by using *in vivo* or *in vitro* bovine systems. By performing an intestinal loop surgical technique, Aich et al. (4) investigated the innate immune responses against bovine BRV in newborn calves. BRV (field isolate BRV85) challenge was able to induce accumulation of fluid and visible histological alterations in the gut of infected animals. Moreover, transcriptional profile of gene expression analysis and qPCR revealed that BRV enhanced TLR3, NF-κB p65, and IL-6. In addition, IRF1, a transcriptional regulator involved in the activation of IFN responses, was activated after rotaviral challenge (4).

We demonstrated that 10-days old BIE cells have developed microvilli-like structures on their surface (45). These characteristics of BIE cells together with their capacity to respond to TLR3 activation allowed us to hypothesize that this cell line could be a valuable *in vitro* tool for studying the interactions between BRV and bovine IECs. Therefore, we compared the infection capacity of four rotavirus strains in BIE cells including human (Wa), murine (EW), porcine (OSU), and bovine (UK), and we found that BIE cells can be effectively infected with the four rotavirus strains (45). Our results showed that 3-day-old BIE cells were more resistant to rotavirus infection than 10-day cultured cells, which probably related to the differences in the length and number of microvilli present on their surfaces. As we mentioned previously, the presence of these cellular structures is important for rotaviral infection since it was suggested that differentiated non-dividing mature enterocytes express the factors that are essential for the efficient rotavirus infection and replication (46, 47). In addition, we found significant differences regarding the viral titers when rotaviruses of different origins were compared. BIE cells were highly infected by bovine and porcine strains, whereas human and murine rotavirus showed a lower capacity to infect this bovine cell line (45). We also observed that viral titers were higher in BIE cells infected with UK than OSU strains, confirming that BRV strain isolated from cattle has a higher capacity to infect these cells. This is in line with previous studies that reported that the infection of porcine small intestinal epithelial (IPEC-J2) cells with OSU rotavirus induced a higher cytopathic effect and significantly reduced cell survival when compared with Wa strain (48). Moreover, our results in BIE cells are also in agreement with our results in porcine IECs (PIE cells) that showed a higher capacity of OSU rotavirus to infect those cells when compared to UK, Wa, or EW strains (49).

**Figure 1:**
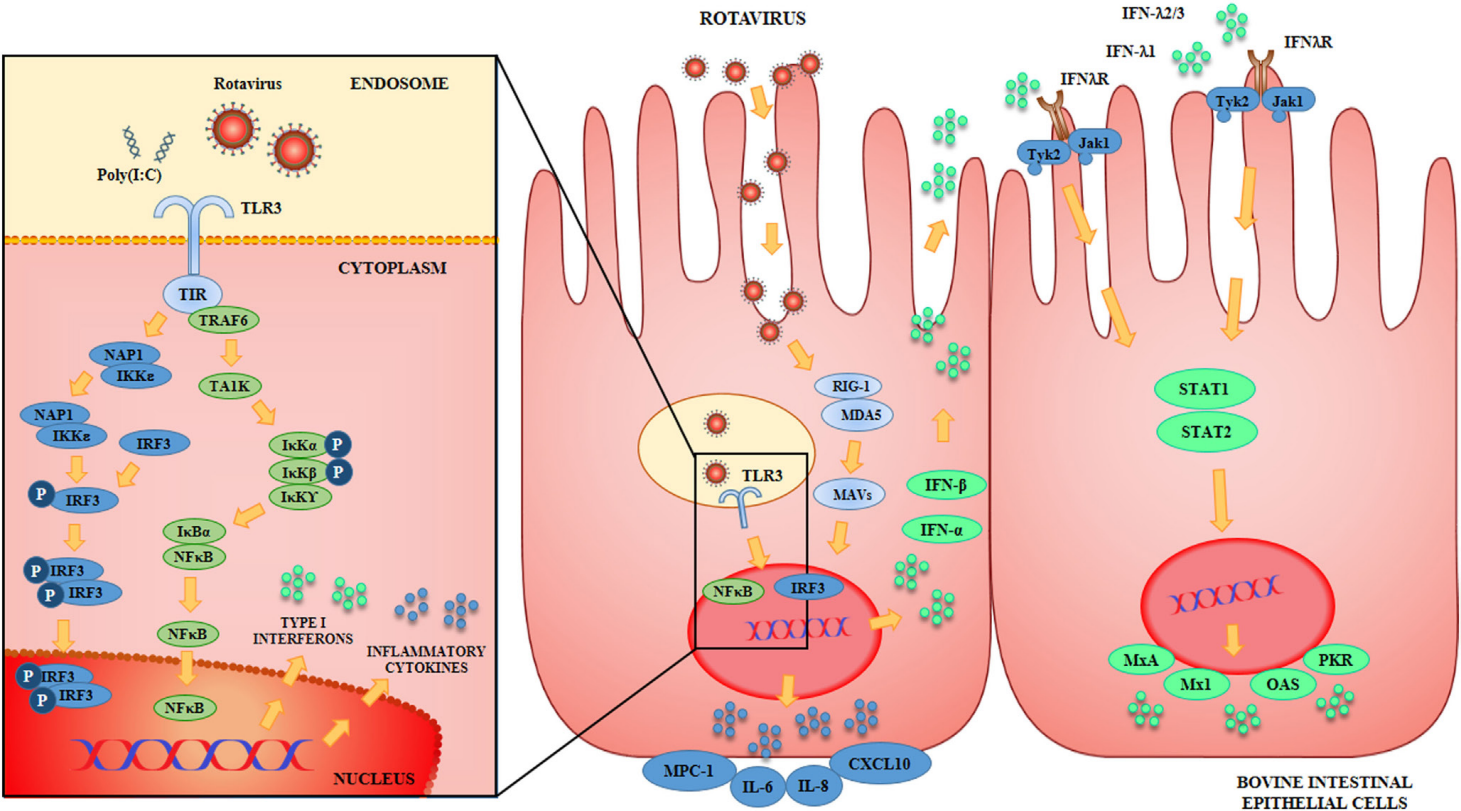
Antiviral Innate immune response against rotavirus in bovine intestinal epithelial (BIE) cells. Rotavirus double-stranded genomic RNA activates toll-like receptor 3 (TLR3), retinoic acid inducible gene-I (RIG-I), and melanoma differentiation-associated gene-5 (MDA-5), which are pattern recognition receptors (PRRs) expressed in BIE cells. Cellular signaling cascades are activated and converge at the level of interferon (IFN) regulatory factor-3 (IRF3) that upregulate the expression of type I (IFN-α, IFN-β) and type III (IFNλ1, IFNλ2/3) IFN, which in turn induces the synthesis of IFN-stimulated genes with antiviral activities including: myxovirus resistance 1 IFN-inducible protein (Mx1), MxA, ribonuclease L (RNaseL), 2′-5′-oligoadenylate synthetase (OAS), and protein kinase R (PKR). Antiviral PRRs also activate nuclear factor κB (NF-κB) pathway and induce the secretion of proinflammatory cytokines and chemokines including: interleukin 6 (IL-6), IL-8, monocyte chemotactic protein 1 (MCP-1/CCL2), and IFN gamma-induced protein 10 (IP-10/CXCL10).

The innate immune response triggered by BRV infection in BIE cells was also characterized (Figure 1). We observed that BRV challenge activated antiviral PRRs in BIE cells and induced immune responses characterized by IFN regulatory factor-3 (IRF3) and NF-κB activation, with the subsequent upregulation of IFN-β and inflammatory chemokines and cytokines. Those results are in agreement with the innate immune mechanisms described for BRV infection in several experimental models as mentioned previously (4, 35, 36). Of interest, we also observed that UK rotavirus was able to induce a stronger innate immune response in BIE cells than OSU strain as demonstrated by the higher levels of expression of inflammatory factors IL-6, IL-8, MCP-1, and IFN-β.

The, BIE cells have several characteristics that make them extremely interesting for the study of BRV pathogenesis, and the molecular mechanisms involved in the generation of innate immune responses. In addition, this cell line could be of value for the evaluation of treatments aimed to beneficially modulate antiviral defenses and reduce inflammatory-mediated damage.

## Bovine IECs as a Model to Select and Characterize Immunobiotics with Antiviral Activity

**Figure 2:**
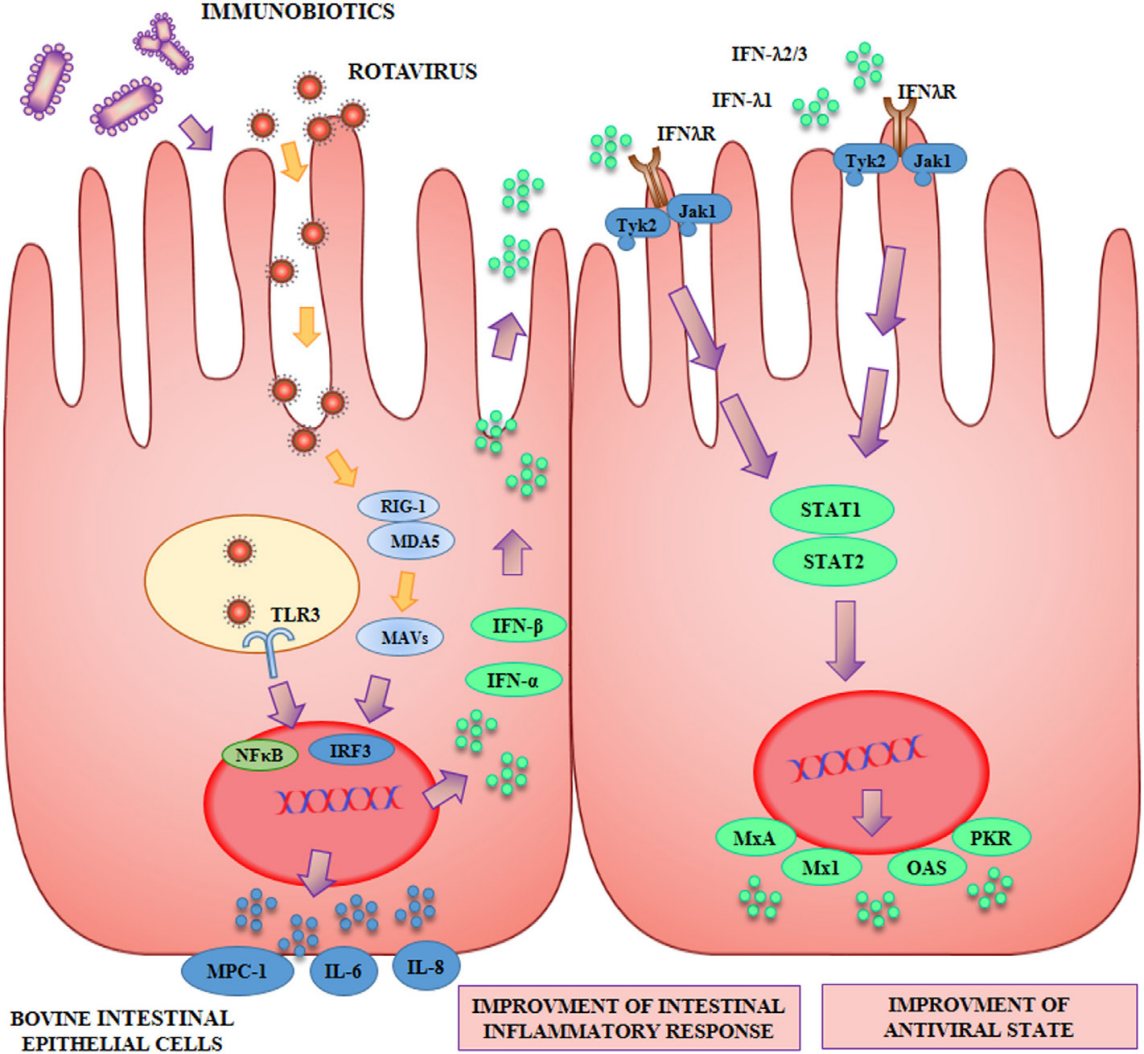
Beneficial effects of immunobiotics on the antiviral innate immune response against rotavirus in bovine intestinal epithelial (BIE) cells. Rotavirus doublestranded genomic RNA activates toll-like receptor 3 (TLR3), retinoic acid inducible gene-I (RIG-I), and melanoma differentiation-associated gene-5 (MDA-5), which are pattern recognition receptors (PRRs) expressed in IECs. Cellular signaling cascades mediated by interferon (IFN) regulatory factor-3 (IRF3) upregulate the expression of type I (IFN-α, IFN-β), and type III (IFNλ1, IFNλ2/3) IFN, which in turn induces the synthesis of IFN-stimulated genes with antiviral activities including: myxovirus resistance 1 IFN-inducible protein (Mx1), MxA, ribonuclease L (RNaseL), 2′-5′-oligoadenylate synthetase (OAS), and protein kinase R (PKR). Antiviral PRRs also activate nuclear factor κB (NF-κB) pathway and induce the secretion of proinflammatory cytokines and chemokines including: interleukin 6 (IL-6), IL-8, monocyte chemotactic protein 1 (MCP-1/CCL2), and IFN gamma-induced protein 10 (IP-10/CXCL10). Preventive treatment of BIE cells with immunobiotics increase the activation of IRF3, improve the production of the antiviral factors and differentially regulate the expression of inflammatory mediators.

The capacity of beneficial microbes to differentially modulate the response of BIE cells to TLR3 stimulation was evaluated by using several lactobacilli and bifidobacteria strains (42, 45) (Figure 2). Some strains such as *L. rhamnosus* LA-2, *S. thermophilus* TMC1543 (42), *B. infantis* MCC12, and *B*. *breve* MCC1274 (45) were able to enhance IFN-β levels after poly(I:C) challenge. The improved production of IFN-β by BIE cells after TLR3 activation induced by those probiotic strains may have significant *in vivo* effects in the protection against enteric viruses. It is well known that IFN-α and IFN-β are important factors of the innate immune response against viral infections. Type I IFNs, after their interaction with the IFN-α/β receptor (IFNAR), upregulate the expression of hundred of antiviral proteins capable to reduce or inhibit viral replication and promote viral clearance. In this regard, transcriptomic analyses of bovine intestinal tissues after the challenge with BRV or BCV have shown that the expression of several IFN-regulated genes is reduced, supporting the conclusion that both viruses have developed mechanism(s) to inhibit immune responses mediated by IFNs (4). Moreover, it has been reported that BVDV is able to impair the induction of type I IFN, which not only affect innate immunity, but in addition interferes with the appropriate development of adaptive immune defenses (5, 50). Based on these findings, immunobiotics that enhance IFN-β production in BIE cells could have a prominent role in the reinforcement of innate and adaptive immune responses against bovine intestinal virus.

As the duration and intensity of proinflammatory factors secretion after TLR3 activation by viral dsRNA can become harmful to the host (51), we also evaluated the levels of key inflammatory cytokines and chemokines in BIE cells including IL-6, IL-8, and MCP-1. Our results indicated that BIE cells pretreated with the probiotic strain *L. casei* TMC0409 (42) produced lower levels of MCP-1, IL-6, and IL-8 when compared with control cells after stimulation with poly(I:C). It has been well established that the unregulated activation of TLR3 is capable to mediate detrimental inflammatory responses in the intestine, thus contributing to the tissue damage induced by viral infections (6, 52). Therefore, the diminished production of proinflammatory factors after the exposure to immunobiotics may allow a better control of the inflammatory responses and reduce the tissue injury mediated by this mechanism. In this way, beneficial bacteria like the TMC0409 strain may offer a different protection mechanism against bovine viral infection.

In conclusion, the BIE cells *in vitro* system was of value for the efficient screening of two types of immunomodulatory probiotic strains capable to improve protection against viral intestinal diseases in the bovine host: (i) strains with the ability to increase antiviral defenses like *L. rhamnosus* LA-2, *B. infantis* MCC12 and *B*. *breve* MCC1274 and (ii) strains with anti-inflammatory capacities like *L. casei* TMC0409.

Considering the mentioned results and that some recent research indicated that the presence of bifidobacteria in the gut of young calf is associated with a good health status (53), we next aimed to evaluate the capacity of the selected immunobiotic bifidobacteria strains to improve resistance of BIE cells to viral challenge.

Studies with the porcine IPEC-J2 cell line, demonstrated that *L. rhamnosus* GG reduced the mucin and IL-6 secretion response triggered by porcine rotavirus, diminishing the inflammatory damage (48). Our own studies in PIE cells also demonstrated that selected immunobiotic strains were capable to upregulate IFN-β expression in response to poly(I:C) stimulation (54, 55). Moreover, we recently demonstrated that *B. infantis* MCC12 and *B. breve* MCC1274 were capable to significantly improve the resistance of PIE cells to porcine rotavirus infection (49). Both immunobiotic bifidobacteria strains significantly enhanced the expression of IFN-β, MxA and ribonuclease L (RNaseL) in infected PIE cells, reducing viral replication. Therefore, we aimed to determine whether the studies performed in our laboratory using porcine IECs could be reproduced in BIE cells. Then, we evaluated whether immunobiotic *B. infantis* MCC12 and *B*. *breve* MCC1274 were able to protect BIE cells against BRV infection.

We showed that BIE cells treated with bifidobacteria were more resistant to BRVs infection, and that MCC12 and MCC1274 treatments significantly increased IFN-β in BRV-infected BIE cells. This is in line with the observation that BRV replication is restricted in susceptible cells by preventive treatment with recombinant IFN-β (56). Likewise, administration of recombinant IFN-β to newborn calves prior to BRV challenge suppresses virus replication and diminishes disease severity (57). A recent study reported the antiviral activity of bacterial strains in mice and Caco-2 cells, in which the probiotic *B. longum* SPM1206 and *L. ruminis* SPM0211 induced the expression of IFN-β in response to human rotavirus (58). Moreover, these probiotic strains had the capacity to inhibit RVs infection through the increase IFN signaling component (STAT1), and IFN-inducible antiviral effectors MXA, protein kinase R (PKR), and OAS in mice. In line with these findings, our recent immunotranscriptomic analysis showed that both *B. infantis* MCC12 and *B*. *breve* MCC1274 are able to improve the expression of several antiviral factors through their capacity to improve IFN-β production. Both bifidobacteria improved the expression of IFN-α and IFN-β as well as the antiviral factors RNASEL, MX1, and MX2 when compared to controls. In addition, bifidobacteria increased the expression of NLRP3 [Albarracin et al. (59), *in preparation*]. In agreement with the central role of IFN-β in the protection of BIE cells against BRV, we also observed that *B*. *breve* MCC1274 induced an earlier and higher activation of TRAF3, higher levels of IFN-β and significantly lower titers of BRV in infected BIE cells when compared with *B. infantis* MCC12. The mechanism(s) (probiotic molecules and host receptors and signaling pathways) by which these immunobiotic bifidobacteria induce higher expression of IFN-β in BRV-infected BIE cells is an interesting topic for future research.

## Conclusion

Prophylactic administration of low doses of antibiotics has been historically used to promote the growth and avoid infectious diseases in livestock animals. However, due to the emergence of antibiotic resistant microbes, several governments in countries around the world have prohibited the use of antibiotics as growth promoters for animals.

One of the most important challenges of agricultural immunology therefore is to find alternatives for developing drug-independent safe food production systems by modulating the immune system of animals. The work reviewed here encourages the research of probiotics to beneficially modulate the immune system of the bovine host. This review provides comprehensive information on the innate antiviral immune response of bovine IECs against virus, which can be further studied for the development of strategies aimed to improve antiviral defenses. The analyzed data also suggest that beneficial microbes have a great potential to be used as antiviral alternatives able to reduce severity of infections in the bovine host.

The development of specific *in vitro* study systems for cattle such as BIE cells as well as the selection and characterization of microbes that exert beneficial functions specifically and efficiently in the bovine host are key points for the successful development of immunomodulatory feeds aimed to protect against infections and reduce or avoid the use of antibiotics.

## Author Contributions

All the authors contributed equally to the design, writing, and editing of the review article.

## Conflict of Interest Statement

The authors declare that the research was conducted in the absence of any commercial or financial relationships that could be construed as a potential conflict of interest.
